# Influence of 5-Week Snack Supplementation with the Addition of Gelatin Hydrolysates from Carp Skins on Pro-Oxidative and Antioxidant Balance Disturbances (TOS, TAS) in a Group of Athletes

**DOI:** 10.3390/antiox11071314

**Published:** 2022-06-30

**Authors:** Małgorzata Morawska-Tota, Łukasz Tota, Joanna Tkaczewska

**Affiliations:** 1 Department of Sports Medicine & Human Nutrition, Faculty of Physical Education and Sport, University of Physical Education, al. Jana Pawła II 78, 31-537 Kraków, Poland; 2 Department of Physiology and Biochemistry, Faculty of Physical Education and Sport, University of Physical Education, al. Jana Pawła II 78, 31-537 Kraków, Poland; lukasz.tota@awf.krakow.pl; 3 Department of Animal Product Technology, Faculty of Food Technology, University of Agriculture, al. Balicka 122, 30-149 Kraków, Poland; joanna.tkaczewska@urk.edu.pl

**Keywords:** antioxidant activity, hydrolyzed proteins, bioactive peptide

## Abstract

The research objective was to assess the effects of 5-week snack supplementation with added enzymatic hydrolysates from carp skins on shifts in pro-oxidative and antioxidant balance among athletes. The study comprised 49 adults (experimental group (E)—17, placebo (P)—16, control (C)—16) practicing endurance disciplines. Selected somatic indices and maximal oxygen uptake/m (VO_2_max) were measured. Based on VO_2_max, an individual exercise intensity was selected with predominating eccentric contractions (60% VO_2_max). The conducted tests consisted of 2 series (1st—graded and eccentric, 2nd—eccentric). The experimental group consumed a snack with added gelatin hydrolysates from carp skins for 5 weeks in between the series, the placebo—a snack without added hydrolysates, and in the control—no supplementation was implemented. Blood samples were taken before, and 1, 24 and 48 h after completion of the eccentric test. TAS and TOC concentrations in the blood plasma were assessed. No significant changes in TOS/TOC and TAS/TAC concentrations were noted between the 1st and the 2nd test series, before or following the eccentric test in the control and placebo groups. In the measurements performed 1, 24 and 48 h after completion, the observed differences were highly significant (*p* < 0.001). After 5 weeks of snack consumption, an increase from medium to high antioxidant potential was observed for E. Differences between the 1st and the 2nd test series were of high statistical significance (*p* < 0.001). The demonstrated differences in pro-oxidative-antioxidant balance indices between successive series allow to confirm antioxidant effects and indicate possibilities for its implementation, not only in sports.

## 1. Introduction

A well-suited diet, in addition to proper nutrition, should increase the rate of regeneration after physical exercise. Along with the increase in exercise intensity, the intensity of respiration processes also experiences an increase. Furthermore, the consumption of oxygen by the skeletal muscles increases, leading to the formation of reactive oxygen species [1]. Certain amounts are necessary for the proper course of intercellular signaling, immune reactions, ensuring the proper functioning of cells, apoptosis and gene transcription [2]. In turn, they play an important role in the physiological adaptation of the body to increased physical activity, as well as cell signaling in the case of athletes [2,3,4], including muscle regeneration and activity of transcription factors [5]. However, the state of their overproduction is defined as oxidative stress and determines disturbed balance between the action of free radicals and the body’s ability to neutralize them. Such an excess of free radicals causes damage to intercellular communication pathways as well as intensification of inflammatory processes [6]. A significant factor influencing redox status is the female sex hormone. Endogenous female sex hormones change during the follicular and luteal phases of the menstrual cycle. Then, 3 significant changes in the ratio of progesterone to estradiol take place [7]. Cyclical changes of these hormones have been correlated with the biological variability of selected oxidative stress markers [8,9], inflammation [10] and other cardiometabolic biomarkers [11]. Moreover, it has been reported that the use of oral contraceptives containing exogenous sex-related steroids may also induce changes in redox homeostasis [12]. Women using combined monophasic oral contraception compared to those using natural contraception have higher resting rates of lipid oxidative damage (i.e., lipid peroxidation) and C-reactive protein inflammation [12]. Moreover, the increased production of reactive oxygen species and the occurrence of intracellular inflammatory processes often occur at a similar time as regeneration and/or adaptation to a physical effort [13]. Thus, potential exposure to increased oxidative stress and CRP, as a result of the active use of oral hormonal contraceptives, may be particularly important during periods of vigorous training or competition, when female athletes are often exposed to additional psychological stress. In order to protect the body against oxidative stress, both endogenous (enzymatic and non-enzymatic) and exogenous antioxidants are first used [14,15]. An important element of modern sport seems to be supporting exercise through the application of various methods and means aimed at accelerating post-workout regeneration and protecting the joints, as well as supplementing energy, micro- and macro-element losses [16,17,18]. Supplements are popular products, and in recent years, their market has been dynamically developing.

Recently, bioactive peptides have received close scientific attention for their broad bioactive scope, mainly including antioxidation, antihypertension, anticancer effects and antimicrobial properties. Antioxidant peptides are especially prominent for their notable contributions to the improvement of human health through the prevention and treatment of non-communicable chronic degenerative diseases, such as cardiovascular and cerebrovascular diseases, cancer, rheumatoid arthritis or diabetes. In addition, peptides with antioxidant properties exert effective metal ion (Fe^2+^/Cu^2+^) chelating activity and lipid peroxidation inhibitory capacity, which endows them with properties that can be potentially used as food-processing additives. The most attractive feature of peptides is their ability to display very few side effects in humans due to their natural sources [19].

In previous research, the authors developed a technology for the production of a gelatin hydrolysate from carp (*Cyprinus carpio*) skins having strong antioxidant properties [20]. As a result, it was found that the high solubility of the obtained hydrolysate within a wide pH range does not limit its use in the food industry. Furthermore, it can be used for food products subjected to heat treatment at temperatures up to 100 °C without adversely affecting their antioxidant properties [21]. Trials among laboratory animals allowed to show that the obtained hydrolysate affected neither liver or kidney function, nor the blood counts of healthy Wistar rats. Therefore, it was concluded that this preparation is safe for usage in living organisms. A statistically significant increase in glutathione reductase activity and total oxidative status of the blood serum in healthy animals fed with the hydrolysate addition is a promising indicator of its in vivo antioxidant properties [22,23].

The various characteristics of chemical structure, namely, low molecular weight, amino acids (His, Trp, Phe, Pro, Gly, lys, Ile and Val) with hydrophobicity, indole/imidazole/pyrrolidine ring, along with the steric structure at the C- and N-termini and the neighboring amino acids of some residues, all indicate some influence on the antioxidant activity of peptides. Among them, the composition and sequence of amino acids have the most impact on antioxidant activity [19]. In our previous study, it was demonstrated that the hydrolysate of carp skin gelatin and its reversed-phase chromatography fractions have strong in vitro antioxidant properties [24]. Among these fractions, the alanine-tyrosine (Ala-Tyr) dipeptide was identified as a major compound with high antioxidant potential. The peptide has good stability during in vitro enzymatic digestion assay and can inhibit the angiotensin-converting enzyme (ACE). In this research, it was proved that both the unfractionated hydrolysates of carp skin gelatin and the above-mentioned Ala-Tyr dipeptide represent attractive novel compounds for the formulation of antioxidant foods.

Thus, it seemed justified to develop a product that could be used in pre-workout nutrition as a source of energy (derived in appropriate proportions from individual nutrients) while supporting the reduction of oxidative stress. This product includes carp meat and a gelatin hydrolysate from carp skin with antioxidant properties. Therefore, the aim of the study was to assess the impact of 5-week snack implementation, with the addition of an enzymatic hydrolysate from carp skins having antioxidant properties, on the shift in pro-oxidative-antioxidant balance among a group of physically active people.

## 2. Materials and Methods

### 2.1. Study Design

The study comprised 49 athletes (experimental group—17, placebo—16, control—16) practicing endurance disciplines (mainly long-distance running). The inclusion criteria for the clinical trial were as follows: gender: male; age: 18–40 years; undertaking regular physical activity; endurance or endurance-strength disciplines; training experience of min. 4 years; participation in sports competitions; no chronic diseases; no contraindications for taking up physical activity. The participants included in the study were informed about the need to stop taking supplements that may affect their level of physical fitness and biochemical indices 6 weeks prior to the first tests. The subjects were enrolled in the study at the end of the transition period of their annual training cycle. The main part of the study covered the preparatory period. The research was approved by the Bioethical Committee at the Regional Medical Committee (No. 205/KBL/OIL/2016). Before beginning the clinical trial, all participants were familiarized with the study protocol (purpose, methodology, benefits, risks) and voluntarily signed an informed consent form with a clause regarding the protection of personal data.

As part of the research project, a 2nd series of research was planned, separated by a 5-week period of supplementation. During this period, participants from the experimental group consumed snacks with the addition of gelatin carp skin hydrolysates, the control group received a snack without the addition of hydrolysates, while the placebo group did not receive any form of supplementation. The subjects were randomly assigned to the above-distinguished groups, and the athletes from the placebo and experimental groups did not know to which group they were designated.

As part of the 1st testing series, somatic measurements, physiological tests (incremental and eccentric exercise tests) and biochemical measurements were planned. As part of the 2nd series of tests, somatic measurements, eccentric exercise test and biochemical measurements were conducted.

During the 1st series of the study, a nutritional consultation was carried out with each athlete to standardize their diet and plan a similar amount of products having sources of antioxidants (350–400 g of vegetables, 250–300 g of fruit).

### 2.2. Somatic Measurements

Measurements of selected somatic indicators were performed twice (1st and 2nd series of tests). The study included measurements of body mass (Tanita 545, Tokio, Japan), body height (SECA 213, Hamburg, Germany) and body structure, performed using the AKERN BIA 101 body composition analyzer (CE0051 certificate, MDD 93\2EEC directive in the area of medical devices, Montacchiello, Piza, Italy).

The average body height and mass of the subjects from each group were similar. A decrease was noted in the mean percentage of body fat in the 2nd series among all study groups, the highest being in the placebo group (Table 1).

### 2.3. Incremental Exercise Test

The graded test was carried out on a treadmill at the Central Laboratory of Science and Research (ISO 9001:2015 in the field of scientific research). The tested effort began with a 4-min warm-up, during which the subject ran at a constant speed of 8 km·h^−1^, at a treadmill inclination angle of 1°. Then, every 2 min, the running speed was increased by 1.0 km·h^−1^. The test was carried out until refusal due to extreme fatigue. The purpose of the test used was to determine the maximal and threshold values corresponding to VT2 (level of second ventilator threshold = respiratory compensation point), as well as the load for the eccentric exercise test (load 60% VO_2_peak). The level of VT2 was verified on the basis of change dynamics in selected indices of the respiratory system. The following criteria for determining the threshold were adopted: ventilatory equivalent for oxygen, ventilatory equivalent for carbon dioxide, minute ventilation, and the percentage of carbon dioxide in exhaled air [25,26]. The highest recorded value was considered the VO_2_peak value.

The highest, maximal speed during the test was recorded in the experimental group (16.4 ± 1.7 km/h), while the lowest was noted in the control group (15.2 ± 2.0 km/h). The maximal oxygen uptake relative to body mass was 55.4 mL/min/kg in the control group, mL/min/kg in the experimental group and 50.7 mL/min/kg in the placebo group, respectively (Table 2).

### 2.4. Eccentric Exercise Test

The test characterized by predominant eccentric contractions was performed by the subjects twice (1st and 2nd series), the purpose of which was to induce muscle damage. During eccentric exercise, muscle extension occurs despite the contraction induced by the central motor drive [27]. Increased muscular pain, tenderness, edema and an efflux of intramuscular proteins into the blood stream are also outcomes of eccentric exercise [28]. During the test, the treadmill was inclined to −10% and the speed was adjusted so that it corresponded to 60 ± 2% of VO_2_peak. To increase muscle cell damage, participants were loaded with rucksacks and metal weights accounting for 5% of their body mass to the nearest 0.1 kg [29]. Metal weights were stabilized in the backpack so as not to disturb co-ordination and balance during the run. Oxygen uptake was continuously monitored in participants by using an ergospirometer to make sure that exercise intensity was equal to 60 ± 2% of VO_2_peak.

All running efforts was performed on the Saturn 250/100R treadmill from h/p/Cosmos (Nussdorf, Traunstein, German)—with an adjustable belt speed and platform angle. During the exercise test, heart rate was measured using the “Polar S 610 i” device (Kempele, Finland). Respiratory indices were recorded using an ergospirometer (Cortex MetaLyzer R3, Leipzig, Germany).

### 2.5. Biochemical Analysis

Blood samples for the determination of biochemical indices were taken from the ulnar vein 60 min prior to testing (T1 in the 1st series; T5 in the 2nd series), with a predominance of eccentric contractions, and 1 h (T2 in the 1st series; T6 in the 2nd series), 24 h (T3 in the 1st series; T7 in the 2nd series) and 48 h (T4 in the first series; T8 in the second series) after the test. The blood sampling procedure was performed in accordance with the applicable standards. Venous blood samples were taken from the participants using the BD Vacutainer System (Becton Dickinson, Franklin Lakes, NJ, USA) and put into tubes containing EDTA as the anticoagulant (for plasma).

Blood plasma samples were obtained by centrifuging the collection tubes for 10 min at 2000 rpm in a MPW 350R laboratory centrifuge (MPW, Warsaw, Poland). Then, they were frozen and stored until analysis at −80 °C (Arctico ULF 390 PRC, Esbjerg, Denmark). All analyses were carried out using the same biochemical tests and apparatus.

In the plasma, using the colorimetric method, the following were determined: TAS/TAC total antioxidant status (Immundiagnostik AG ImAnOx, test sensitivity: 130 µmol·L^−1^), and TOS/TOC total pro-oxidative status of the plasma (test by Immundiagnostik AG PerOx, sensitivity of 7 µmol·L^−1^). 

Due to post-exercise dehydration, the values of biochemical indices determined after completing the eccentric test were corrected. Corrections were made by first calculating %ΔPV from the formula [30], while the formula according to Kraemer and Brown (1984) [31] was used to calculate the corrected values. 

### 2.6. Analysis of Nutrition

Both before the eccentric test during the 1st and 2nd series, the athletes kept food diaries (the day before the test, the day of the test, the day after the test) using the current recording method in order to quantify the consumption of regulating ingredients with antioxidant activity (vitamins: A, E, C; minerals: zinc, copper). The obtained nutritional data were analyzed using ‘Tables’ regarding the composition and nutritional value of food [32]. Following this stage, they were compared to the recommendations of the Food and Nutrition Institute [33].

### 2.7. Tested Snack

The snack being the subject of the study included carp meat, as well as, inter alia, products demonstrating an alkalizing effect with low and medium glycemic load. Carp meat comprised 29%, dried dates 36%, buckwheat flakes 25%, pecan nuts 3.25%, sunflower seeds 2.25%, water 3% and cinnamon in the amount of 0.5%. In bars intended for the experimental group, 1% of the enzyme additive of hydrolysate from carp skin was included and described in previous studies [20,22,34]. In the snacks intended for the placebo group, the applied hydrolysate was water. During the supplementation period (5 weeks), athletes from the placebo and experimental groups ate 1, 70 g-bar a day as part of post-workout nutrition, and on days off from training, they consumed the bar at any time of the day. The period of supplementation for athletes from the experimental and placebo groups started after the final blood sampling procedure in the 1st series and ended after the 3rd blood sampling intervention during the 2nd series. Detailed characteristics of the recipe, snack production technology and NOAEL (No-observable-and-adverse-effect-level) and ADI (Acceptable Daily Intake) dose determination are presented in the publication by Tkaczewska et al., 2021 [34].

### 2.8. Statistical Analysis

Analysis was performed with the IBM SPSS Statistics package, version 26.0.0.0, using two-way analysis of variance in a mixed model. A separate analysis of variance was performed for each of the 3 groups (experimental, placebo and control). First, the assumption about sphericality of variance was tested, and if it was met, classic two-factor analysis of variance was performed. If this assumption was not met, the Greenhouse–Geisser correction was applied. The homogeneity of variance and the equality assumption of the covariance matrix using the Box test were also checked via Levene’s test.

In each of the cases, the inter-subject effect between the 2 series was tested, and for TAS/TAC, this effect for each of the 2 series of tests was also verified. The test probability was assumed to be significant at the level of *p* < 0.05, and highly significant at *p* < 0.01.

## 3. Results

### 3.1. Nutrition

It was shown that the athletes from all 3 groups provided proper amounts of the analyzed regulating ingredients with antioxidant properties [33]. The supply of the analyzed vitamins and minerals in both series was similar within the individual groups (Table 3). 

### 3.2. Prooxidative and Antioxidant Status

**Figure 1:**
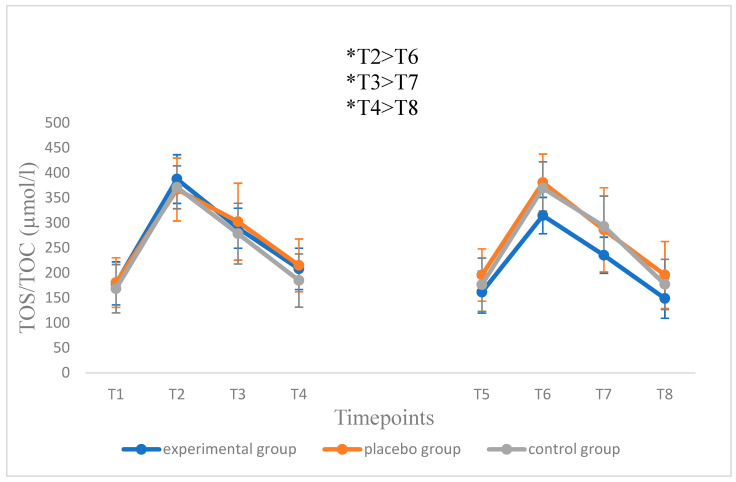
The level of TOS/TOC index in all of the tested groups during the 1st (T1–T4) and 2nd (T5–T8) series of testing. Blood sampling: T1 = 1 h before eccentric test in the 1st series; T2 = 1 h; T3 = 24 h; T4 = 48 h following the eccentric test in the 1st series (beginning of 5-week supplementation period in the experimental and placebo groups); T5 = 1 h before the eccentric test in the 2nd series; T6 = 1 h; T7 = 24 h; T8 = 48 h after the eccentric test in the 2nd series of testing (end of supplementation). Data are presented as mean values ± standard deviation. Significant differences (*p* < 0.05) were indicated at respective time-points for the experimental (*), placebo (^#^) and control groups (^+^).

A characteristic increase in the TOS/TOC ratio 1 h after exercise was observed, followed by its gradual decrease in the control (*p* = 0.799) and placebo groups (*p* = 0.926). There were no significant changes in the concentration of TOS/TOC between the 1st and 2nd series, both before (control group 168.3 vs. 176.3; placebo 180.5 vs. 195.6) and after the eccentric test. In the control group, after the completion of the test in the 1st series, the values ranged from 371.0 (1 h after exercise) to 184.6 µmol/L (48 h after its completion), and in the 2nd series, from 369.3 to 176.9 µmol/L. In the placebo group, these values were 366.7–214.9 vs. 380.7–195.8 µmol/L. In the experimental group, a similar tendency was observed only before beginning the test. In the measurements performed 1, 24 and 48 h after its completion, the differences were highly statistically significant (*p* < 0.001) (Figure 1).

**Figure 2:**
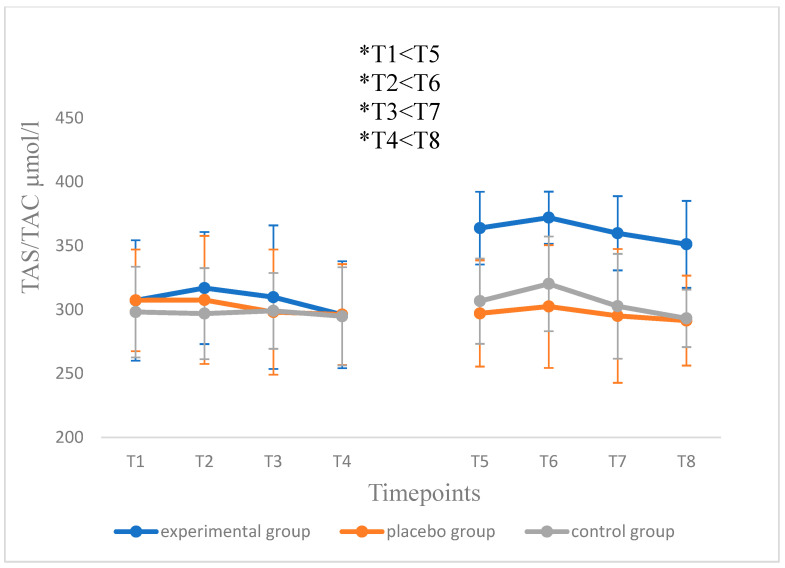
Level of TAS/TAC index in all of the tested during the 1st (T1–T4) and 2nd (T5–T8) testing series. Blood sampling: T1 = 1 h before eccentric test in the 1st series; T2 = 1 h; T3 = 24 h; T4 = 48 h following the eccentric test in the 1st series (beginning of 5-week supplementation period in the experimental and placebo groups); T5 = 1 h prior to the eccentric test in the 2nd series; T6 = 1 h; T7 = 24 h; T8 = 48 h after the eccentric test in the 2nd testing series (end of supplementation). Data are presented as mean values ± standard deviation. Significant differences (*p <* 0.05) were indicated at respective time-points for the experimental (*), placebo (^#^) and control groups (^+^).

No significant differences in the mean plasma antioxidant potential (TAS/TAC) were observed between the 1st and 2nd series during any of the measurements in the control (*p* = 0.297) or placebo groups (*p* = 0.670). In turn, before the beginning of the test with the predominance of eccentric contractions, this potential was at the level of 307.2 ± 47.1 μmol·L^−1^ in the experimental group during the 1st series of tests, and in the second series, 363.9 ± 28.5 μmol·L^−1^, which allowed to indicate that supplementation with the hydrolysate-added snack allowed to increase the antioxidant potential from medium to high. A similar trend was observed in subsequent measurements, and the differences in the mean plasma antioxidant potential between the 1st and 2nd series were of statistical significance (*p* < 0.001) (Figure 2).

## 4. Discussion

### 4.1. Nutrition and Oxidative Stress

Various lifestyle factors, including exercise, have been classified as modulators of oxidative stress induced by reactive oxygen species (ROS). They are mainly released as by-products of mitochondrial oxidative metabolism or as a cell’s defense mechanism in response to xenobiotics, cytokine production and bacterial invasion [35]. Moreover, they are involved in determining the purpose of cells and modulating various pathways (growth, differentiation, progression and cell death) [36]. The production of ROS can negatively affect cells and tissues by inducing lipid and protein peroxidation. Therefore, the increase in the production of free radicals is associated with a decrease in the activity of enzymatic systems responsible for their removal, as well as a decrease in antioxidant activity, causing them to be ineffectively removed by the body’s defense mechanisms. Increased or prolonged oxidative stress may lead to permanent changes in the structure of biologically significant macromolecules (DNA, proteins, sugars, etc.), and furthermore, they can contribute to overtraining [6]. It should be borne in mind, however, that ROS, at physiological concentrations, are important signaling factors that regulate growth, proliferation and differentiation. Their production induced by physical exercise is an important element for inducing biological adaptation to training at tissue and cellular levels (including muscle angiogenesis, mitochondrial synthesis). Therefore, on the one hand, supplementation with antioxidants may disturb balance between free radicals and the endogenous antioxidant mechanism, changing the physiological adaptive responses [37]. On the other, low consumption or reduced bioavailability of antioxidants from food weakens the antioxidant network, contributing to the occurrence of oxidative stress. In the available literature, a negative impact has been shown of antioxidant supplementation on exercise capacity, expression of adaptive genes and protein synthesis, which may be a consequence of weakening the redox homeostasis pathway in muscles [38]. Thus, it seems justified to optimize and adjust antioxidant supplementation to dietary supply, as well as the exact redox status, type of exercise and muscle fatigue [14]. In our own research, we indicated the correct intake of vitamins and minerals with antioxidant properties among all subjects for both series of testing, therefore, this factor should not affect the level of pro-oxidative and antioxidant status among the participants. In turn, in the research by Devrim-Lanpir et al. (2020), total antioxidant intake in the diet of ultra-endurance athletes was negatively correlated with pre-exercise TOS concentrations while, at the same time, being positively correlated with ∆TAS concentrations. A positive and significant (*p* = 0.013) interaction was observed between exhaustion time and antioxidant consumption (rs = 0.692) in the group of males. Such a relationship was not found among women [3].

### 4.2. Effect of Fish Protein Hydrolysate on Pro-Oxidative Status

Fish industry waste is of high biological value, and its hydrolysates mainly contain di- and tripeptides with much better digestibility than unchanged free amino acids and proteins [39]. Also demonstrated has been their beneficial effect on the cardiovascular and central nervous systems, the intestines, kidneys and the immune system [40], as well as a strong antioxidant influence [41,42,43]. The sequence and activity of bioactive peptides with different origins have been fairly well-researched, but in only a few studies has their activity been assessed in a human model. Moreover, the results are often disappointing. Those obtained in vitro and in animal models are promising, however, their transfer to the human model is often not confirmed. The emerging discrepancies may be influenced, among others, by the fact that for in vitro models, the industrial production process as well as the digestive and metabolic steps are frequently ignored. In addition, doses used in vitro and administered to laboratory animals are often higher than those administered in the case of humans [44].

The exact mechanism underlying the antioxidant activity of peptides has not been fully understood, yet, in various studies, it has been stated that they are inhibitors of lipid peroxidation, scavengers of free radicals and chelators of transition metal ions. In addition, it has been reported that antioxidative peptides keep cells safe from damage by ROS through the induction of genes [45]. Furthermore, according to Erdmann et al. 2006, the Met-Tyr dipeptide from sardine muscle prevents oxidative stress by stimulating expression of heme oxygenase-1 (HO-1) and ferritin (antioxidant defense proteins) in endothelial cells [46]. Moreover, results from another study revealed that protein hydrolysates could enhance the activities of glutathione peroxidase (GSH-Px) and superoxide dismutase (SOD), reducing malondialdehyde (MDA) concentration in vivo [47]. 

In the research by Lu et al. (2020) [48], the effect of supplementation with fish protein hydrolysates was evaluated in a mouse model during extreme physical exertion until refusal. A significant increase in resistance to fatigue induced by the physical effort and an extension of swimming time was demonstrated in the groups among which the supplementation was used (carp polypeptides 120 min vs. 27 min–control group). These results correspond to those obtained in the authors’ study, indicating a reduction in oxidative stress after supplementation with the snack having added gelatin hydrolysates from carp skins. On the other hand, different results were obtained with the use of marine protein hydrolysates (MPH) in combination with whey protein (WP) and carbohydrates (CHO) compared to supplementation with WP + CHO. Supplementation with the addition of marine protein hydrolysates did not have a significant effect on short-term regeneration in the group of healthy, middle-aged males subjected to high-intensity cycling sessions [49].

### 4.3. Supplementation and Antioxidant Potential of the Athletes’ Plasma

Supplements are popular products and in recent years, their market has been developing very dynamically. According to estimates by Grand View Research, their global value in 2019 amounted to USD 123.28 billion, and forecasts indicate a further increase in the coming years. A constantly changing lifestyle, including greater interest in physical activity and rational nutrition, has caused a greater demand for products with a potentially beneficial effect on health and physical fitness. Despite the relatively low level of knowledge about various substances distributed in the form of supplements, people are using them more and more often [50,51,52,53], while their inappropriate consumption, especially those substances synthetically obtained, may be detrimental to health [51,54]. In numerous studies, the effect of supplementation with antioxidant substances on the antioxidant potential of athletes, its efficiency and regeneration has been assessed. Research has been conducted with the use of, among others, vitamins C and E [55], sour cherry juice [56], pomegranate juice [57], cocoa [58,59], green and/or sour tea [60,61] and acai berry pulp [62]. In literature on the subject, the strong antioxidant effect of protein hydrolysates from the fish industry is often emphasized [41,42,43]. However, there are no studies in which it would be shown what effect their use has on the antioxidant potential of human plasma. In this study, a significantly higher antioxidant potential (TAC) was reported among the participants who consumed a snack with the added carp skin hydrolysate for 5 weeks. Similar results were obtained by Urbaniak et al. (2018) [57] after supplementation with pomegranate juice among a men’s rowing team, noting a significant increase in TAC value; as well as by Hadi et al. (2017) [60] in a group of football players after 6 weeks of supplementation with 450 mg of green and sour tea.

## 5. Conclusions

Evaluating effectiveness regarding the application of bioactive peptides cannot be based solely on in vitro studies and animal models. In order to confirm their applicative value, it seems necessary to conduct research among humans. The presented results indicate great potential of enzymatic hydrolysates from fish industry waste, both for the environment and for reducing oxidative stress among individuals professionally and recreationally performing sports. It should be emphasized that the level of physical capacity in all of the studied groups was similar, while only in the experimental group were there significant differences in the pro-oxidative-antioxidant balance indices observed between subsequent series. These changes allow to confirm the antioxidant effect of the developed product and indicate possibilities of its use other than just in sports.

## 6. Patents

Patents resulting from the work reported in this manuscript: No. P.424604 granted by the Patent Office of the Republic of Poland.

## Figures and Tables

**Table 1:** Somatic features of the study participants.

SERIES	BH [cm]	BM [kg]	FFM [kg]	FM [%]
control group x ± SD				
I	180.7 ± 7.2	75.9 ± 6.9	66.7 ± 5.1	12.0 ± 4.1
II	180.7 ± 7.2	75.8 ± 6.5	66.8 ± 5.0	11.7 ± 4.2
experimental group x ± SD				
I	181.0 ± 4.9	74.0 ± 5.6	61.3 ± 4.0	17.1 ± 3.1
II	181.0 ± 4.9	73.8 ± 5.8	61.2 ± 4.3	16.8 ± 2.8
placebo group x ± SD				
I	179.1 ± 5.3	75.6 ± 11.1	63.1 ± 8.1	16.2 ± 4.4
II	179.1 ± 5.3	75.7 ± 11.6	64.3 ± 8.3	14.7 ± 5.1

Data are expressed as mean ± SD; BH—body height; BM—body mass; FFM—fat-free mass; FM—fat mass; I—1st series (befor supplementation –experimental and placebo group); II—2ndseries (after supplementation-experimental and placebo group).

**Table 2:** Mean values of exercise (threshold and maximal) level regarding selected physiological indices in particular groups.

	Index →	t [min]	v [km∙h^−1^]	HR [sk∙min^−1^]	VO_2_ [L∙min^−1^]	VO_2peak_ [mL·min^−1^·kg^−1^]	Ve [L∙min^−1^]
Level ↓							
control group x ± SD							
VT2		10.5 ± 2.5	11.4 ± 1.5	168.6 ± 9.1	3.4 ± 0.6	45.2 ± 8.7	92.1 ± 13.6
max		17.0 ± 2.5	15.2 ± 2.0	188.3 ± 14.0	4.2 ± 0.6	55.4 ± 8.7	151.5 ± 22.4
experimental group x ± SD							
VT2		11.3 ± 2.0	12.0 ± 1.7	164.5 ± 9.1	3.3 ± 0.4	45.1 ± 5.5	95.0 ± 18.7
max		18.3 ± 1.8	16.4 ± 1.7	186.1 ± 8.9	4.0 ± 0.3	54.9 ± 4.9	157.5 ± 14.6
placebo group x ± SD							
VT2		11.6 ± 1.9	12.5 ± 1.3	170.0 ± 7.7	3.1 ± 0.4	42.1 ± 7.1	93.6 ± 17.3
max		17.5 ± 3.7	16.1 ± 2.5	188.6 ± 8.4	3.8 ± 0.5	50.7 ± 9.5	152.7 ± 24.8

Data are expressed as mean ± SD; VT2—level of second ventilatory threshold; max—maximal exercise level; t—test duration; v—run speed; HR—heart rate; VO_2_—maximal oxygen uptake in global terms; VO_2_max—maximal oxygen uptake per minute relative to body mass; Ve—minute respiratory ventilation.

**Table 3:** Average supply of regulating ingredients with antioxidant properties in relation to the norms in the 3 studied groups.

Component	Vitamin A [µg]		Vitamin E [mg]		Vitamin C [mg]		Zinc [mg]		Copper [mg]	
Series	I	II	I	II	I	II	I	II	I	II
control group	898.9 ± 193.8	888.5 ± 173.9	10.5 ± 0.8	10.4 ± 0.5	102.2 ± 18.8	98.3 ± 14	11.2 ± 1.7	11.2 ± 1.4	1.3 ± 0.4	1.3 ± 0.4
experimental group	984.1 ± 198.9	987.3 ± 161.0	10.7 ± 1.0	10.8 ± 1.0	132.6 ± 49.9	125.2 ± 42.1	11.0 ± 1.2	10.7 ± 1.3	1.4 ± 0.3	1.4 ± 0.3
placebo group	936.8 ± 241.9	938.4 ± 240.1	11.5 ± 1.5	11.1 ± 1.2	127.6 ± 52.9	125.4 ± 60.4	11.7 ± 2.5	11.4 ± 2.2	1.3 ± 0.4	1.2 ± 0.4
Norm AI/EAR	630		10		75		9.4		0.7	

Data are expressed as mean ± SD; I—1st series (before supplementation—experimental and placebo group); II—2nd series (after supplementation- experimental and placebo group).

## Data Availability

Data is contained within the article.
